# Back Propagation Artificial Neural Network Enhanced Accuracy of Multi-Mode Sensors

**DOI:** 10.3390/bios15030148

**Published:** 2025-02-26

**Authors:** Xue Zou, Xiaohong Wang, Jinchun Tu, Delun Chen, Yang Cao

**Affiliations:** 1 State Key Laboratory of Marine Resource Utilization in South China Sea, College of Material Science and Engineering, Hainan University, Haikou 570228, China; 22210805000026@hainanu.edu.cn (X.Z.); tujinchun@hainanu.edu.cn (J.T.); 2 School of Chemistry and Chemical Engineering, Hainan University, Haikou 570228, China; wangxiaohong@hainanu.edu.cn; 3 College of Science, Qiongtai Normal University, Haikou 571100, China

**Keywords:** BP ANN, multi-mode sensor, PB

## Abstract

The detection of small molecules is critical in many fields, but traditional electrochemical detection methods often exhibit limited accuracy. The construction of multi-mode sensors is a common strategy to improve detection accuracy. However, most existing multi-mode sensors rely on the separate analysis of each mode signal, which can easily lead to sensor failure when the deviation between different mode results is too large. In this study, we propose a multi-mode sensor based on Prussian Blue (PB) for ascorbic acid (AA) detection. We innovatively integrate back-propagation artificial neural networks (BP ANNs) to comprehensively process the three collected signal data sets, which successfully solves the problem of sensor failure caused by the large deviation of signal detection results, and greatly improves the prediction accuracy, detection range, and anti-interference of the sensor. Our findings provide an effective solution for optimizing the data analysis of multi-modal sensors, and show broad application prospects in bioanalysis, clinical diagnosis, and related fields.

## 1. Introduction

Accurate determination of small molecule concentrations in body fluids is critical for individual health assessment, early disease screening, and optimization of treatment options. For example, high blood glucose concentration is often a precursor to diabetes [1,2,3,4], while excessive cholesterol levels can significantly increase the risk of atherosclerosis [5,6]. Additionally, lack of ascorbic acid (AA) predisposes to scurvy [7,8,9,10,11], and elevated blood levels of homocysteine are associated with a heightened risk of cardiovascular and cerebrovascular diseases [12,13,14,15]. Electrochemical detection has emerged as one of the most commonly used methods for measuring small molecule concentrations due to its high sensitivity, wide detection range, fast response, and low cost [16]. However, many small molecules exhibit structural and chemical similarities; for example, the redox potentials of dopamine, uric acid, and ascorbic acid are very close to each other [17], which often leads to poor detection accuracy.

Most studies focus on the development of multi-mode sensors to improve the sensing performance. For example, Yue Hu et al. constructed a dual-mode sensing platform for electrochemiluminescence and colorimetry detection, which improves selectivity by carefully designing the cathode and anode to achieve an accurate prediction of glucose concentration [18]. Aixiang Liu et al. designed a high-efficiency peroxidase simulated nanozyme for dual-mode sensors, enabling high-precision detection of AA [19]. Lei Peng et al. developed an electrochemical and colorimetric dual-mode sensing platform based on copper/zirconium metal–organic framework (Cu/Zr-MOF) nanozymes with high peroxidase-like activity to achieve highly sensitive detection of H_2_O_2_ [20]. While multi-mode sensors can effectively improve detection accuracy, many of them still rely on the individual analysis of each signal, and the data utilization is insufficient, which can lead to potential sensor failure when results from different modes are biased greatly.

Back-propagation artificial neural networks (BP ANNs), are a powerful data processing tool capable of processing both quantitative and qualitative information; they are good at coordinating the relationship between different input data, and have been widely used in a variety of fields such as performance prediction, target detection, medical diagnosis, autonomous driving, and mechanical fault diagnosis [21]. In addition, the BP ANN is particularly effective in dealing with complex, uncertain, or unknown systems, as they can automatically extract features from the input data through learning and training, without manually specifying rules. Its strong approximation ability to nonlinear functions, combined with robustness to minor variations in the input data, and tolerance for a certain degree of noise and errors in input, thus improves the anti-interference ability and prediction accuracy [22,23].

Therefore, in order to improve the accuracy of small molecule concentration detection, this study constructs a high-performance multi-modal sensor by selecting appropriate materials, and focusing on the comprehensive analysis of multi-mode signals. This approach distinguishes our work from the conventional multi-modal sensors, which typically analyze each mode independently. In our method, the BP ANN is applied to multi-mode sensors to achieve accurate concentration measurement under strong interference conditions. We constructed an electrochemical/ultraviolet multi-mode sensor based on PB for AA detection. The sensor simultaneously processes signals from open-circuit voltage (OCV), chronoamperometry (CA) obtained by electrochemical measurements, and the intensity signal of ultraviolet (UV) absorption peak obtained from a UV spectrophotometer. The results showed that the analysis results of the BP ANN are more accurate than those of each modality independently, significantly improving the prediction accuracy and broadening the detection range of the sensor. Therefore, using the BP ANN (implementing using Python 3.12.0).

For data analysis of multi-modal sensors can effectively improve the prediction accuracy and detection range of small molecule detection. These findings provide an effective solution for enhancing the data analysis of multi-modal sensors, and have broad application prospects in biological analysis and clinical diagnosis.

## 2. Materials and Methods

### 2.1. Synthesis of PB, Ni-PB, and Co-Ni-PB

PB was prepared following the method proposed by Uemura Takashi [24]. First, 20 mg FeCl_2_·4H_2_O (0.10 mmol) and 1.11 g PVP (K-30; average molecular weight = 50,000) were added to 8 mL of aqueous solution as solution ①, then 33 mg of K_3_Fe(CN)_6_ (0.10 mmol) was weighed and added to 2 mL of aqueous solution as solution ②. After complete dissolution, solution ② was slowly added to solution ① at room temperature and stirred vigorously. The mixture immediately turned blue after the addition, forming a PB with good water solubility. After 20 min of reaction, 25 mL of acetone was added to precipitate PB, and then the resulting precipitate was centrifuged and washed several times with acetone.

Considering that Fe-based materials often incorporate doped Co and Ni elements to enhance the electron transfer and improve the electron conductivity of the material [25,26], Ni-doped and Co- and Ni co-doped PB were also prepared to improve the performance of the sensor. The preparation method of Ni-PB and Co-Ni-PB was similar to that of the above method, except that 0.01 mM Ni(NO_3_)_2_ was added to solution ① for the synthesis of Ni-PB, and 0.005 mM Ni(NO_3_)_2_ and 0.005 mM Co(NO_3_)_2_ were added to solution ① for the synthesis of Co-NiPB.

### 2.2. Construction and Detection of Electrochemical/Ultraviolet Multi-Mode Sensors

Fluorine-doped tin dioxide (FTO) glass (2 × 1 × 0.22 cm) was successively sonicated in glass cleaning agent and ultrapure water for 20 min. Subsequently, the obtained PB was prepared into an aqueous solution of a certain concentration, 100 μL was evenly dropped on the surface of the FTO electrode, and dried in an oven at 60 °C for 24 h, and then evenly covered with 100 μL of 1% Nafion solution on the surface for another 24 h to ensure that the PB on the electrode would not dissolve during the electrochemical test.

The OCV and CA signals of different concentrations of AA were obtained by electrochemical measurement in a 0.1 M KCl solution using the above electrodes. And the UV signals of different concentrations of AA were obtained by testing the mixed solution of PB and AA in a cuvette with a UV spectrophotometer, where the PB concentration in each mixed solution was the same, but the AA concentration was different.

### 2.3. Construction of BP ANN

The BP ANN model consists of three major components: the input layer, the hidden layer, and the output layer. In this study, the input layer of the BP ANN includes three nodes, corresponding to three detection signals: OCV, CA, and UV. Among them, OCV takes the stable voltage value, CA takes the stable current value, and UV takes the absorbance peak value. And the BP ANN has two hidden layers, each of which is designed with three nodes. Because there is no linear correlation between the sensor signals of each mode within all concentration ranges, it is appropriate to select three nodes in the BP neural network, sufficient to prevent overfitting from affecting the reliability of the BP neural network. The Sigmoid function is used as the activation function in both the hidden layer and the output layer. The result obtained by the output layer is the predicted concentration of AA.

In addition, the learning rate and the number of epochs are also two important parameters of the BP ANN model. A learning rate that is too high may prevent the model from converging and hinder the discovery of an optimal solution, while a rate that is too small can lead to too slow training or fall into the local optimum. Similarly, an excessively high number of epochs can easily lead to overfitting and increased cost, while too few epochs may result in underfitting and inaccurate predictions. After debugging, a learning rate of 0.9 and epochs of 5 × 10^4^ were selected for this study. Under this condition, the mean square error is used as the loss function, and the parameters of the neural network are continuously adjusted to reduce the value of the loss function, thereby improving the prediction effect of the model.

Finally, a total of 17 groups of valid data were obtained, and 14 of them were selected as the training set, while all 17 sets were used for the validation and test sets.

### 2.4. Instruments

Scanning electron microscopy (SEM) was performed with a Sigma 300 field emission scanning electron microscope from Zeiss, Oberkochen, Germany. Transmission electron microscopy (TEM) was performed with a JEOL JEM2100 instrument in Tokyo, Japan. X-ray diffraction (XRD) patterns were obtained on the Smart Lab SE X-ray diffractometer in Rigaku, Tokyo, Japan. X-ray photoelectron spectroscopy (XPS) was tested with a k-alpha from Thermo Fisher, Waltham, MA, USA, and calibrated by C 1s (284.8 eV). The Raman instrument was a Thermo Fisher DXR2xi (Waltham, MA, USA), and the Fourier Transform Infrared Spectroscopy (FTIR) instrument was a BRUKER T27 (Karlsruhe, Germany). The electrochemical tests were performed on the Biologic VMP-300 electrochemical workstation from Bio-Logic, France, using a three-electrode system, i.e., a saturated calomel electrode as the reference electrode, platinum wire as the counter electrode, and an FTO glass loaded with the material as the working electrode. UV-Vis absorption spectroscopy was tested on Hitachi’s UH5700 UV-Vis near-infrared spectrophotometer.

## 3. Results and Discussion

### 3.1. Characterization of PB

**Figure 1:**
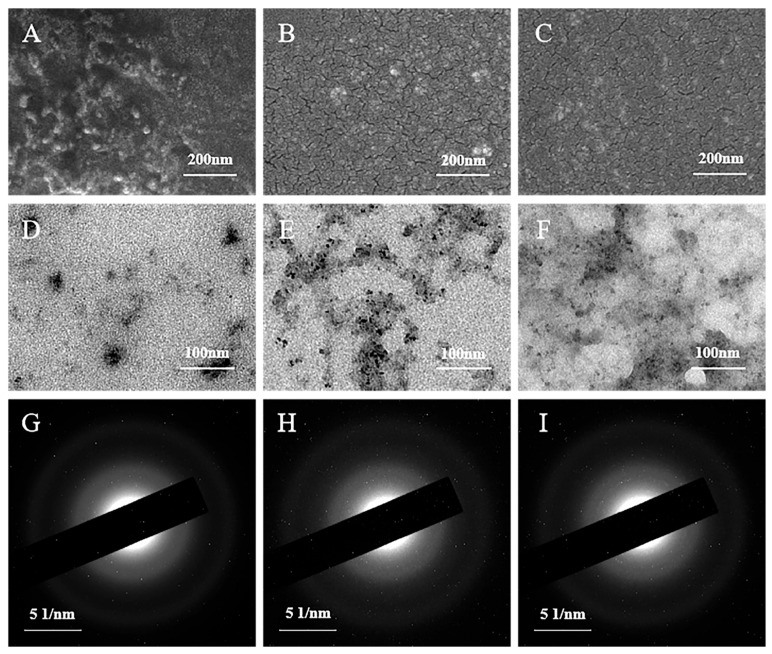
(**A**–**C**) SEM images, (**D**–**F**) TEM images, and (**G**–**I**) electron diffraction patterns of PB, Ni-PB, and Co-Ni-PB.

First, the morphological changes in PB before and after doping were examined by scanning electron microscopy (SEM) and transmission electron microscopy (TEM). As shown in Figure 1A–C, the SEM images reveal that all three prepared materials are nanoparticles, which enhance their performance. Notably, the sizes of the doped Ni-PB and Co-Ni-PB particles are smaller, and their dispersion is improved. This is probably due to the influence of Co and Ni on the nucleation and growth process of the grains [27], which refines the grain structure and subsequently enhances the electrochemical effective area and the number of active sites in the materials. The TEM images of Figure 1D–F further illustrate the topography changes before and after doping, which is consistent with the SEM observations. The electron diffraction patterns in Figure 1G–I indicate a relatively poor crystallinity of all three materials, which may affect their stability to a certain extent. In addition, Figure S1 displays the EDS mapping of the three materials, which characterizes their elemental composition and distribution, showing that the Fe, Co, Ni, and N elements are uniformly distributed in the corresponding materials. These results confirm the successful incorporation of Co and Ni elements into PB.

**Figure 2:**
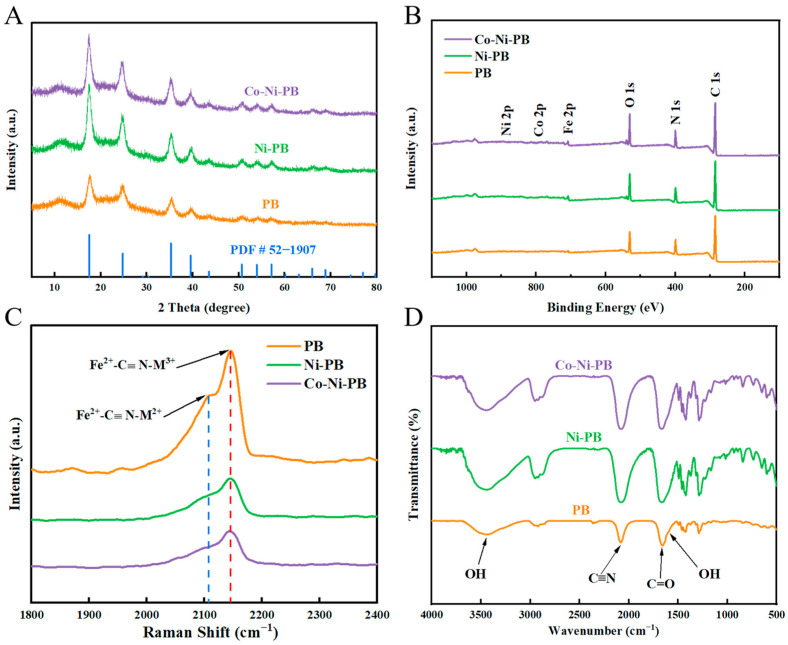
(**A**) XRD pattern. (**B**) Full spectrum of XPS measurements. (**C**) Raman spectrum. The peak corresponding to the dotted blue line belongs to Fe^2+^–CN–M^2+^, and the peak corresponding to the dotted red line belongs to Fe^2+^–CN–M^3+^. (**D**) FTIR spectrum.

Then, the crystal structure and physical phase purity of PB, Ni-PB, and Co-Ni-PB samples were investigated using X-ray diffraction (XRD), as shown in Figure 2A. The XRD patterns of all samples display the characteristic diffraction peaks of PB, with a notable peak at 11.25°, corresponding to the surfactant PVP [28]. No additional peaks were observed, indicating the successful preparation of PB. The enlarged view of the XRD pattern (Figure S2) shows that the peaks corresponding to the (200) crystal planes of Ni-PB and Co-Ni-PB are shifted compared with those of PB, indicating that the introduction of Co and Ni induces a certain degree of lattice distortion, which further proves that Co and Ni elements are successfully incorporated into PB. Then, the chemical compositions and corresponding chemical valences of the PBs before and after Co and Ni doping were examined by XPS, as shown in Figure 2B and Figure S3. The high-resolution XPS spectra (Figure S3) support the presence of Fe ions in both +2 and +3 oxidation states across all three materials, with peaks located at 724.4 and 720.7 eV attributed to Fe^3+^ and Fe^2+^ in Fe 2p_1/2_, and the peaks at 711.7 and 707.9 eV assigned to Fe^3+^ and Fe^2+^ in Fe 2p_3/2_. The Ni ions in Ni-PB mainly existed in the form of Ni^2+^, and both Co and Ni in Co-Ni-PB existed in divalent forms [29,30].

Furthermore, the chemical environment of each cation was investigated using Raman spectroscopy (Figure 2C). The spectra reveal two vibrational peaks at 2107 cm^−1^ and 2146 cm^−1^, which can be attributed to Fe^2+^–CN–M^2+^ and Fe^2+^–CN–M^3+^ [31,32]. The chemical structures of the three materials were further investigated by Fourier transform infrared (FTIR) spectroscopy (Figure 2D). The peak observed at 2081 cm^−1^ is attributed to the typical stretching vibration of the C≡N group, while the peak at 1656 cm^−1^ is attributed to the C=O stretching of the PVP. The weak peak at 1607 cm^−1^ and the broad peak at 3403 cm^−1^ are derived from the –OH bending and stretching vibrations of H_2_O, respectively [24,29]. In short, PB, Ni-PB, and Co-Ni-PB were successfully prepared.

### 3.2. Performance Analysis of Single Signal Based on Electrochemical/Ultraviolet Multi-Mode Sensor

**Figure 3:**
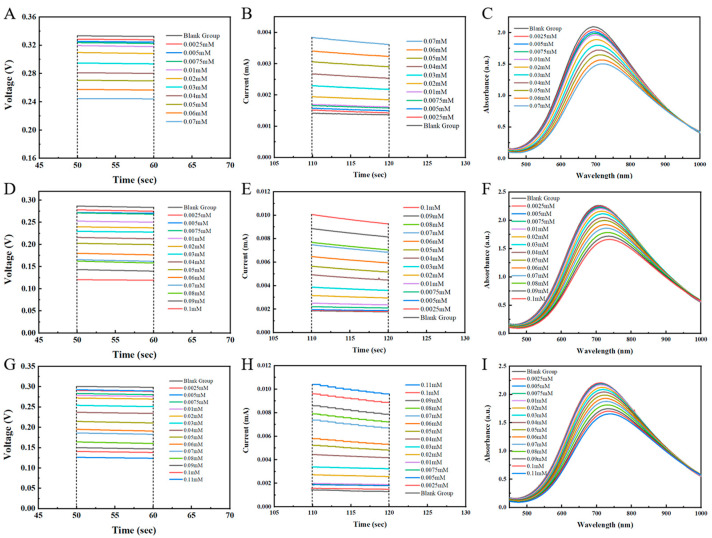
OCV, CA, and UV absorption of (**A**–**C**) PB, (**D**–**F**) Ni-PB, and (**G**–**I**) Co-Ni-PB at different concentrations of AA.

In order to ensure the reliability of the data used for BP ANN analysis, the three prepared materials were used to construct electrochemical/ultraviolet multi-mode sensors, and their sensing performance was explored and compared. Figure 3 demonstrated the OCV, CA, and UV absorption at different AA concentrations, and the results indicate that with the increase in AA concentration, the OCV, CA, and UV absorption signals of these materials changed significantly. Specifically, both the OCV and UV absorption peak intensities decreased with increasing AA concentration, while the CA signal increased with the increase in AA concentration.

As the concentration of AA increases, the decrease in OCV signal may be due to the fact that PB, as a transition metal complex with a unique crystal structure, exhibits excellent redox activity, while AA has certain reducing properties because its molecular structure contains specific hydroxy functional groups [33,34,35,36]. Therefore, when detecting AA, PB will undergo a redox reaction with AA. As the concentration of AA increases, according to the equilibrium principle of chemical reactions, namely the Le Chatelier’s principle, the reaction will proceed in the direction of consuming more AA and generating more reduced state PB, resulting in redox equilibrium on the electrode surface to move toward the reduced state [37,38]. According to the Nernst equation, the electrode potential is closely related to the ratio of the concentration of the oxidized substance and the reduced substance. The concentration of oxidized substances is relatively reduced, and the concentration of reducing substances is relatively increased, which will cause the electrode potential to change. At this time, under open circuit conditions, the change in the electrode potential is directly reflected in the decrease in the open circuit voltage.

And the decrease in UV absorbance of PB with increasing AA concentration may be because PB is a compound with a specific crystal structure and electron cloud distribution in which iron ions are in a specific valence state. In the reaction with AA, some iron ions in PB that were originally in a higher valence state may be reduced to a lower valence state, resulting in changes in their electron cloud distribution. And the ability of matter to absorb ultraviolet light is closely related to its electronic structure. Thus, as the concentration of AA increases, more PB structures change and the concentration of the effective absorbent material decreases, which according to the Lambert–Beer law, causes a decrease in its absorbance at the corresponding UV wavelength [39].

While the increase in CA signal with increasing AA concentration is due to the oxidation reaction of AA on the electrode surface. According to the principle of chemical reaction kinetics, an increase in the reactant concentration will speed up the reaction rate. Thus, as the AA concentration increases, the reaction rate accelerates, and the number of electron transfers through the electrode surface increases per unit time, resulting in a larger oxidative current, namely the CA signal enhancement [37,38].

Figure S4 shows the calibration curves for the three signals versus concentration. As can be seen from the figure, the OCV, CA, and UV absorption signals of the sensor based on PB (Figure S4A–C) exhibit a good linear relationship with AA concentrations in the range of 0–70 μM. The OCV, CA, and UV absorption signals of the Ni-PB sensor (Figure S4D–F) demonstrate a good linear relationship with AA concentration in the range of 0–100 μM. The OCV, CA, and UV absorption signals of the Co-Ni-PB sensor (Figure S4G–I) show the widest linear detection range, with a good linear relationship with AA concentrations in the range of 0–110 μM. And the typical range of ascorbic acid in the bloodstream is approximately 40–80 μM [40], indicating that each sensor has practical application value. The R^2^ values for all modes were greater than 0.99, indicating that each mode had strong predictive ability.

Furthermore, the electrochemical test sensitivity of the Co-Ni-PB sensor is better than that of the PB and Ni-PB sensors. However, the UV test sensitivity of the Co-Ni-PB sensor is slightly lower than that of the PB and Ni-PB sensors. In addition, the confidence bands and prediction bands presented in Figure S4 are crucial for evaluating the reliability of linear regression as well as the accuracy of numerical estimates. In a specific range, the narrower the confidence interval, the more accurate the estimate of the overall parameters and the higher the confidence level. Similarly, a narrower prediction band enhances the accuracy of the mode’s prediction for future observations. It is notable that some of the data points are at the edge of the 95% confidence interval, and the prediction interval is relatively wide (Figure S4), which implies that there is still room for improvement in the accuracy of linear regression in predicting AA concentrations, despite the good fit of linear regression.

**Figure 4:**
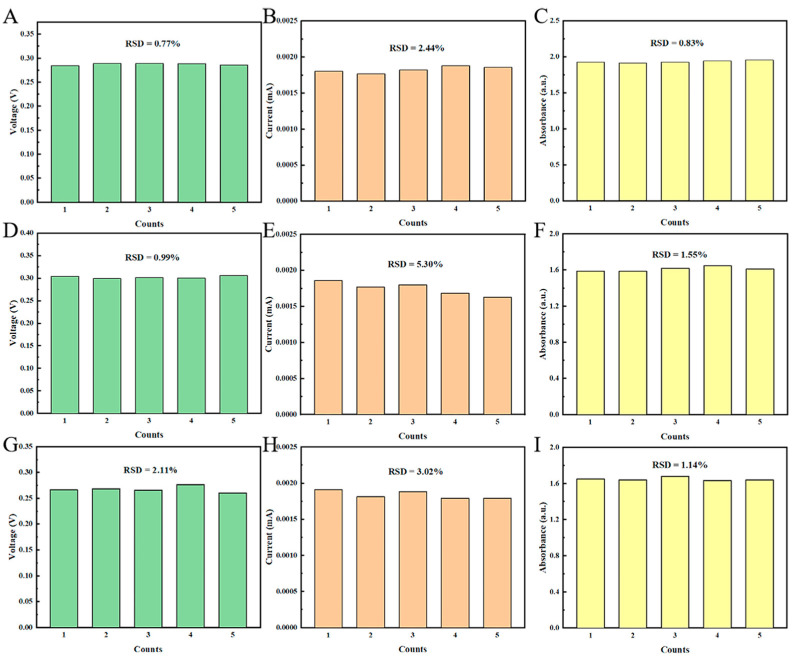
Repeatability testing of OCV (Green bar chart), CA (Orange bar chart), and UV absorption (Yellow bar chart) from sensors prepared by (**A**–**C**) PB, (**D**–**F**) Ni-PB, and (**G**–**I**) Co-Ni-PB, respectively.

Furthermore, in order to evaluate the repeatability of each sensing mode (Figure 4), five replicate experiments were conducted. The results showed relatively poor repeatability of the CA test for each sensing mode, and good repeatability of the OCV and UV absorption tests. Among them, the overall repeatability of the PB sensor is the best, which may be due to the slight decrease in material stability caused by the lattice distortion after doping of Co and Ni atoms.

**Figure 5:**
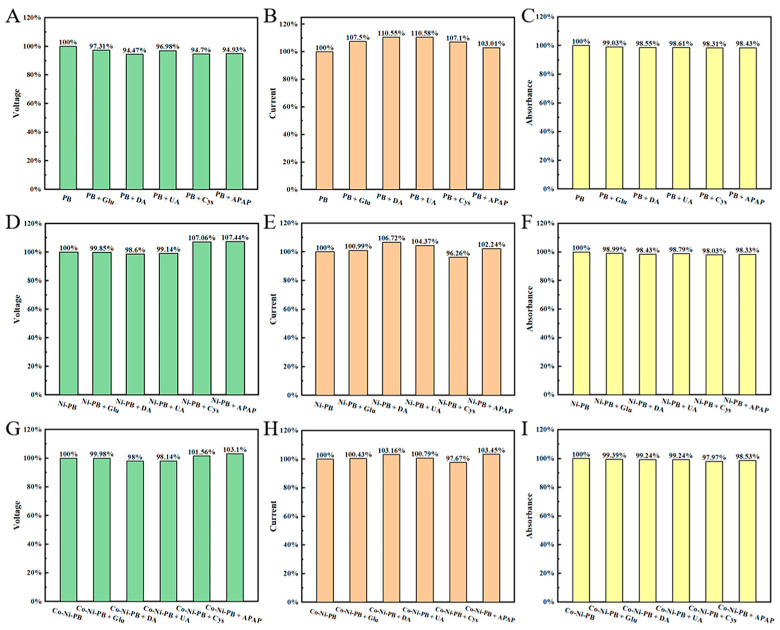
Selective testing of OCV (Green bar chart), CA (Orange bar chart), and UV (Yellow bar chart) absorption from sensors prepared by (**A**–**C**) PB, (**D**–**F**) Ni-PB, and (**G**–**I**) Co-Ni-PB, respectively. The concentration of AA in each group was 0.05 mM, and the concentration of each interference was 0.005 mM.

Finally, to evaluate the anti-interference performance of each sensing platform, the selectivity of each sensor for AA in the presence of uric acid (UA), dopamine (DA), glucose (Glu), acetaminophen (APAP), and cysteine (Cys) was also tested, as shown in Figure 5. The results show that the overall selectivity is ideal, with the Co-Ni-PB sensor demonstrating optimal selectivity across all modes. However, the selectivity of the CA mode of each sensing mode is worse than that of the other two.

Overall, the Co-Ni-PB sensor has the widest linear detection range for OCV, CA, and UV absorption signals, which means it can detect AA over a broader concentration range. And its sensitivity is also optimal, enabling more precise detection of AA concentration changes. Although its stability is slightly inferior to that of the PB sensor but better than that of the Ni-PB sensor, it shows the best selectivity in all detection modes. Considering these factors together, the Co-Ni-PB sensor is considered to have the optimal comprehensive performance. These multi-mode sensors are all able to realize mutual verification by dissecting the detection results in each mode individually, which effectively improves the reliability of the detection results. However, it should be noted that the test performance of the CA mode is relatively suboptimal. In the case of strong interference factors, the test results obtained from this mode may have a significant deviation from the other two modes, complicating the final judgment of the test results. This situation underscores the necessity of using the BP ANN.

### 3.3. Comprehensive Analysis of Data Based on BP ANN

Based on the discussions above, we selected the data obtained from the Co-Ni-PB sensor for the BP ANN analysis. In addition, a few more concentrations were measured as shown in Figure S5, and the results showed that the multi-mode sensor had a good linear relationship between signal and concentration only in the range of 0~0.11 mM. Generally, when the detection error of the sensor is less than 5%, the prediction result is acceptable. However, it can be seen from Table 1 that the prediction accuracy of each model is unsatisfactory, especially at lower concentrations.

To address this, the three signal data points of the Co-Ni-PB sensor were input into the constructed BP ANN model for calculation. To ensure the accuracy of the calculation results, each concentration data was calculated for 50 replicates (Figure S6), and the average value was taken as the prediction result from the BP ANN. The results show that the prediction results of the BP ANN are very close to the actual value, including the detection range higher than 0.11 mM, which significantly improves the detection range. Additionally, the RSD of each figure indicates that the stability of BP ANN is good.

**Figure 6:**
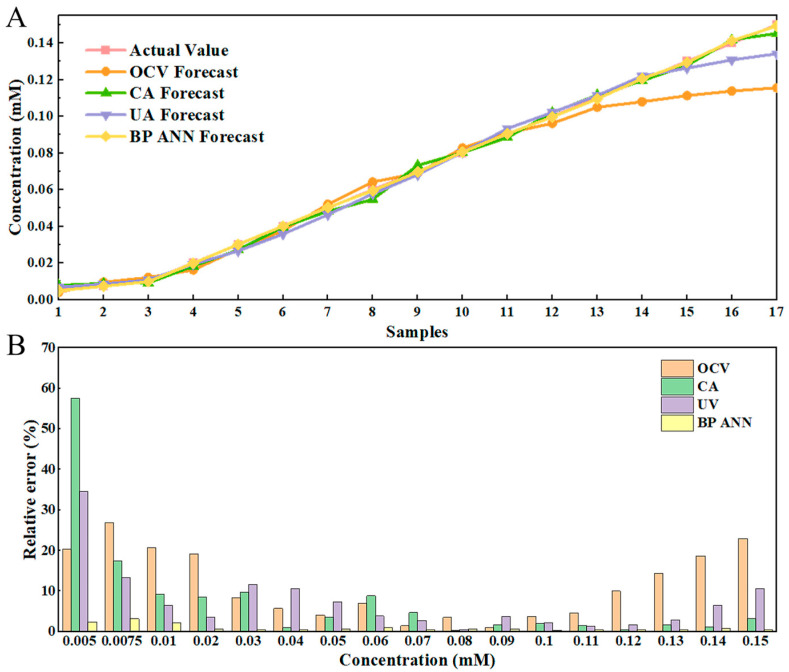
(**A**) The prediction results, and (**B**) the errors of OCV, CA, and UV and the BP ANN at different concentrations.

**Figure 7:**
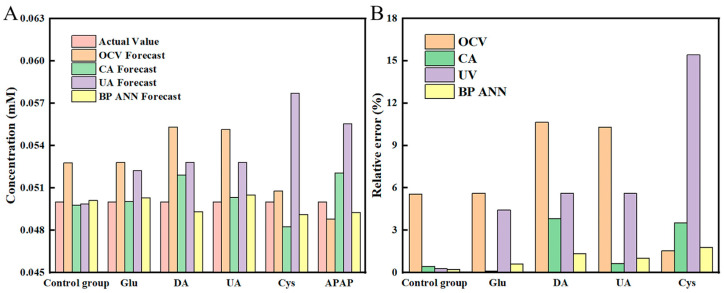
(**A**) The prediction results and (**B**) the errors of OCV, CA, and UV, and the BP ANN under different interferences. The concentration of AA in each group was 0.05 mM, and the concentration of each interference was 0.005 mM.

Compared with the prediction results of the OCV, CA, and UV models, the BP ANN model showed obvious superiority, as its predicted results are significantly closer to the actual concentration of AA (Figure 6A and Figure 7A). More importantly, the prediction error of the BP ANN for each concentration was consistently below 5%, which was notably lower than that of the OCV, CA, and UV modes (Figure 6B). Even when the estimation error of the OCV, CA, and UV model test exceeded 50%, the error of the BP ANN remained below 3.5%, highlighting its robustness and excellent generalization ability. Moreover, in the presence of interference factors, even if the prediction error of one of the modes is greater than 15%, the BPANN can still achieve accurate prediction and ensure that the error is less than 2%, highlighting the strong fault tolerance of the BP ANN (Figure 7B). The above results show that the BP ANN can extract accurate concentration data from the analysis of low-quality detection results, demonstrating good prediction performance, which significantly improves the detection accuracy and anti-interference ability of the sensor.

## 4. Conclusions

In conclusion, the multi-mode sensor based on Co-Ni-PB demonstrates the best overall performance, but its test performance still exhibits relative shortcomings in the CA mode, which is prone to significant data fluctuations. These fluctuations can interfere with the accurate judgment of the final test results. The prediction accuracy of single signal output from each single mode also needs to be further improved. To address these issues, the BP ANN was introduced to comprehensively process the signal data collected by the three sensing modes. This innovative approach effectively mitigates the risk of sensor malfunction resulting from significant deviations in the detection results of a particular signal. The predictions generated by the BP ANN are more accurate than the isolated analysis of each sensing mode, which significantly improves the prediction accuracy of the sensor, broadens the detection range, and improves the anti-interference performance. These breakthrough findings have opened up an effective new way to optimize the data analysis process of multi-modal sensors, and are expected to show broad and far-reaching applications in cutting-edge fields such as bioanalysis and clinical diagnosis, thereby injecting strong impetus into the development of related fields.

## Figures and Tables

**Table 1:** Prediction error for the different modes.

Concentration (mM)	OCV (%)	CA (%)	UV (%)
0.005	20.32	57.48	34.64
0.0075	26.90	17.44	13.29
0.01	20.67	9.25	6.53
0.02	19.16	8.58	3.61
0.03	8.31	9.69	11.56
0.04	5.76	0.91	10.63
0.05	4.07	3.64	7.33
0.06	7.00	8.80	3.83
0.07	1.45	4.67	2.72
0.08	3.48	0.26	0.56
0.09	1.00	1.69	3.76
0.1	3.78	2.10	2.21
0.11	4.56	1.55	1.29

## Data Availability

The data presented in this study are available on request. The code for BP ANN in this study can be found on GitHub (Python 3.12.0). https://github.com/926-Xue/Back-Propagation-Artificial-Neural-Network-Enhanced-Accuracy-of-Multi-mode-Sensors.git (accessed on 17 February 2025).

## Supplementary Materials

The following supporting information can be downloaded at: https://www.mdpi.com/article/10.3390/bios15030148/s1, Figure S1: EDS mapping of (A–C) PB, (D–G) Ni-PB, and (H–L) Co-Ni-PB; Figure S2: The enlarged view of XRD pattern; Figure S3: Fe 2p XPS spectra of (A) PB, (B) Ni-PB, and (C) Co-Ni-PB. Ni 2p XPS spectra of (D) Ni-PB and (E) Co-Ni-PB. Co 2p XPS spectra of (F) Co-Ni-PB; Figure S4. Calibration curves of OCV, CA, and UV absorption of (A–C) PB, (D–F) Ni-PB, and (G–I) Co-Ni-PB; Figure S5: OCV, CA, and UV absorption of Co-Ni-PB under the 0~0.15 mM concentration of AA; Figure S6: 50 prediction results of BP ANN at different test concentrations.

## References

[1] Cole J.B., Florez J.C. (2020). Genetics of diabetes mellitus and diabetes complications. Nat. Rev. Nephrol..

[2] Ajjan R.A., Battelino T., Cos X., Del Prato S., Philips J.-C., Meyer L., Seufert J., Seidu S. (2024). Continuous glucose monitoring for the routine care of type 2 diabetes mellitus. Nat. Rev. Endocrinol..

[3] Narasaki Y., Kalantar-Zadeh K., Daza A.C., You A.S., Novoa A., Peralta R.A., Siu M.K.M., Nguyen D.V., Rhee C.M. (2024). Accuracy of Continuous Glucose Monitoring in Hemodialysis Patients With Diabetes. Diabetes Care.

[4] Gómez-Peralta F., Luque Romero L.G., Puppo-Moreno A., Riesgo J. (2024). Performance of a Non-Invasive System for Monitoring Blood Glucose Levels Based on Near-Infrared Spectroscopy Technology (Glucube^®^). Sensors.

[5] King R.J., Singh P.K., Mehla K. (2022). The cholesterol pathway: Impact on immunity and cancer. Trends Immunol..

[6] Mortensen M.B., Dzaye O., Bøtker H.E., Jensen J.M., Maeng M., Bentzon J.F., Kanstrup H., Sørensen H.T., Leipsic J., Blankstein R. (2023). Low-Density Lipoprotein Cholesterol Is Predominantly Associated With Atherosclerotic Cardiovascular Disease Events in Patients With Evidence of Coronary Atherosclerosis: The Western Denmark Heart Registry. Circulation.

[7] Wang M., He J., Li S., Cai Q., Zhang K., She J. (2023). Structural basis of vitamin C recognition and transport by mammalian SVCT1 transporter. Nat. Commun..

[8] Thiemann S., Cimorelli V., Bajwa N.M. (2022). Case Report: Uncommon cause of limp in the 21st century. Front. Endocrinol..

[9] Lu R.-L., Guo J.-W., Sun B.-d., Chen Y.-L., Liu D.-Z. (2023). Scurvy in a young man: A rare case report. Front. Nutr..

[10] Skerniskyte J., Mulet C., André A.C., Anderson M.C., Injarabian L., Buck A., Prade V.M., Sansonetti P.J., Reibel-Foisset S., Walch A.K. (2023). Ascorbate deficiency increases progression of shigellosis in guinea pigs and mice infection models. Gut Microbes.

[11] de Jong T.M.H., Stamatelou E., Rosema N.A.M., Jansen I.D.C., Brandt B.W., Angelakis A., Loos B.G., van der Velden U., Danser M.M. (2024). Effect of Daily Vitamin C Supplementation with or Without Flavonoids on Periodontal, Microbial, and Systemic Conditions Before and After Periodontal Therapy: A Case Series from an RCT. J. Clin. Med..

[12] Tripathi M., Singh B.K., Zhou J., Tikno K., Widjaja A., Sandireddy R., Arul K., Abdul Ghani S.A.B., Bee G.G.B., Wong K.A. (2022). Vitamin B12 and folate decrease inflammation and fibrosis in NASH by preventing syntaxin 17 homocysteinylation. J. Hepatol..

[13] Fan F., David Spence J., Huo Y. (2023). Beyond hypertension: Hypertension with hyperhomocysteinemia. Sci. Bull..

[14] Ojaroodi A.F., Jafarnezhad F., Eskandari Z., Keramat S., Stanek A. (2025). Recent Updates and Advances in the Association Between Vitamin D Deficiency and Risk of Thrombotic Disease. Nutrients.

[15] Machoń N.J., Zdanowska N., Klimek-Trojan P., Owczarczyk-Saczonek A. (2025). Vascular Cell Adhesion Molecule 1 and E-Selectin as Potential Cardiovascular Risk Biomarkers in Psoriasis. Int. J. Mol. Sci..

[16] Ma X., Gao W., Du F., Yuan F., Yu J., Guan Y., Sojic N., Xu G. (2021). Rational Design of Electrochemiluminescent Devices. Acc. Chem. Res..

[17] Jothi L., Neogi S., Jaganathan S.k., Nageswaran G. (2018). Simultaneous determination of ascorbic acid, dopamine and uric acid by a novel electrochemical sensor based on N2/Ar RF plasma assisted graphene nanosheets/graphene nanoribbons. Biosens. Bioelectron..

[18] Hu Y., Zhu L., Mei X., Liu J., Yao Z., Li Y. (2021). Dual-Mode Sensing Platform for Electrochemiluminescence and Colorimetry Detection Based on a Closed Bipolar Electrode. Anal. Chem..

[19] Liu A., Song W., Zhang C., Shang H. (2024). Colorimetry/Smartphone Dual-Mode Sensing Platform Based on Nanorod-Shaped Ni–Fe MOFs for Ascorbic Acid Detection. ACS Appl. Nano Mater..

[20] Lei P., Wu N., Zhou Y., Dong C., Liu Y., Shuang S. (2024). Cu/Zr Metal–Organic Frameworks with High Peroxidase-Like Activity for Sensitive Electrochemical and Colorimetric Dual-Mode Detection of Hydrogen Peroxide Released from Living Cells. ACS Appl. Nano Mater..

[21] Lin S., Zhou Y., Hu J., Sun Z., Zhang T., Wang M. (2022). Exploration for a BP-ANN model for gas identification and concentration measurement with an ultrasonically radiated catalytic combustion gas sensor. Sens. Actuators B Chem..

[22] Tong A., Tang X., Liu H., Gao H., Kou X., Zhang Q. (2023). Differentiation of NaCl, NaOH, and β-Phenylethylamine Using Ultraviolet Spectroscopy and Improved Adaptive Artificial Bee Colony Combined with BP-ANN Algorithm. ACS Omega.

[23] Wu D., Zhang D., Liu S., Jin Z., Chowwanonthapunya T., Gao J., Li X. (2020). Prediction of polycarbonate degradation in natural atmospheric environment of China based on BP-ANN model with screened environmental factors. Chem. Eng. J..

[24] Uemura T., Kitagawa S. (2003). Prussian Blue Nanoparticles Protected by Poly(vinylpyrrolidone). J. Am. Chem. Soc..

[25] Li B., Wang Y., Huang S., Fang R., Zhang L., Jin Y., Guo K. (2025). Co-doped Ni-PBA anchored on optimized ZIF-67-derived Co/N-doped hollow carbon framework for high-performance hybrid capacitive deionization. Sep. Purif. Technol..

[26] Wang C., Zhang J., Wang L., Feng J., Wang L., Dong L., Long C., Li D., Hou F., Liang J. (2023). Ternary transition metal of Fe/Co/Ni doping on MoSx nanowires for highly efficient electrochemical oxygen evolution. Sustain. Mater. Technol..

[27] Ali S.K., Asghar A., Rashid M.S., Karmouch R., Alathlawi H.J., Syed I.S., Awaji M.Y., Almashnowi M.Y.A., Gulfam Q.-u.-A., Sultana H. (2024). Effects of Co-doping on the microstructure and electrochemical performance of nickel vanadate (Ni_3_V_2_O_8_) electrode material for aqueous symmetric supercapacitor. Inorg. Chem. Commun..

[28] Mosad W., Sherif H.H.A., Hemdan B., Khalil Ibrahim W., Khalil S. (2017). Assessment of in situ-Prepared Polyvinylpyrrolidone-Silver Nanocomposite for Antimicrobial Applications. Acta Phys. Pol. A.

[29] Du M., Geng P., Pei C., Jiang X., Shan Y., Hu W., Ni L., Pang H. (2022). High-Entropy Prussian Blue Analogues and Their Oxide Family as Sulfur Hosts for Lithium-Sulfur Batteries. Angew. Chem..

[30] Hu H., Lin H., Chen X., Pan Y., Li X., Zhuang Z., Chen H., Wang X., Luo M., Zheng K. (2023). Fe, Mn-Prussian blue analogue@MXene composites for efficient photocatalytic peroxydisulfate-activated degradation of tetracycline hydrochloride and photoelectrochemical desalination. Chem. Eng. J..

[31] Wang L., Lu Y., Liu J., Xu M., Cheng J., Zhang D., Goodenough J.B. (2013). A Superior Low-Cost Cathode for a Na-Ion Battery. Angew. Chem. Int. Ed..

[32] Dai J., Tan S., Wang L., Ling F., Duan F., Ma M., Shao Y., Rui X., Yao Y., Hu E. (2023). High-Voltage Potassium Hexacyanoferrate Cathode via High-Entropy and Potassium Incorporation for Stable Sodium-Ion Batteries. ACS Nano.

[33] Wang Y., Liang Z., Liang Z., Lv W., Chen M., Zhao Y. (2022). Advancements of Prussian blue-based nanoplatforms in biomedical fields: Progress and perspectives. J. Control. Release.

[34] Li B., Dai Y., Shi C., Guo X., Chen Y., Zeng W. (2024). Flexible molecularly imprinted glucose sensor based on graphene sponge and Prussian blue. Bioelectrochemistry.

[35] Xu J., Gui M., Li Z., Fang Y., Wang S., Li H., Yu R. (2025). Enhanced colorimetric analysis substrate based on graphene oxide open-close actuator. Sens. Actuators B Chem..

[36] Ricci F., Palleschi G. (2005). Sensor and biosensor preparation, optimisation and applications of Prussian Blue modified electrodes. Biosens. Bioelectron..

[37] Teepoo S., Chumsaeng P., Jongjinakool S., Chantu K., Nolykad W. (2012). A New Simple and Rapid Colorimetric Screening Test for Semi-qualitative Analysis of Vitamin C in Fruit Juices Based on Prussian Blue. J. Appl. Sci..

[38] Castro S.S.L., Balbo V.R., Barbeira P.J.S., Stradiotto N.R. (2001). Flow injection amperometric detection of ascorbic acid using a Prussian Blue film-modified electrode. Talanta.

[39] El Mously D.A., Mahmoud A.M., Abdel-Raoof A.M., Elgazzar E. (2022). Synthesis of Prussian Blue Analogue and Its Catalytic Activity toward Reduction of Environmentally Toxic Nitroaromatic Pollutants. ACS Omega.

[40] Witmer J.R., Wetherell B.J., Wagner B.A., Du J., Cullen J.J., Buettner G.R. (2016). Direct spectrophotometric measurement of supra-physiological levels of ascorbate in plasma. Redox Biol..

