# Supplementary material for: Back Propagation Artificial Neural Network Enhanced Accuracy of Multi-Mode Sensors
Source: Biosensors (Basel). 2025 Feb 26;15(3):148. doi: 10.3390/bios15030148 (PMC11940617; doi:10.3390/bios15030148)
Supplement: Supplementary file 1 [file biosensors-15-00148-s001.zip › biosensors-3467916-supplementary.pdf]

Supplementary Materials

Back Propagation Artificial Neural Network Enhanced Accuracy of Multi-mode Sensors

Xue Zou [1], Xiaohong Wang [2], Jinchun Tu [1], Delun Chen [1,*] and Yang Cao [1,3,*]

[1] State Key Laboratory of Marine Resource Utilization in South China Sea, College of Material Science and Engineering, Hainan University, Haikou 570228, China.

[2] School of Chemistry and Chemical Engineering, Hainan University, Haikou 570228, China.

[3] College of Science, Qiongtai Normal University, Haikou 571100, China.

* Correspondence: chendelun2014@163.com (D. Chen), cy507@hainanu.edu.cn (Y. Cao).

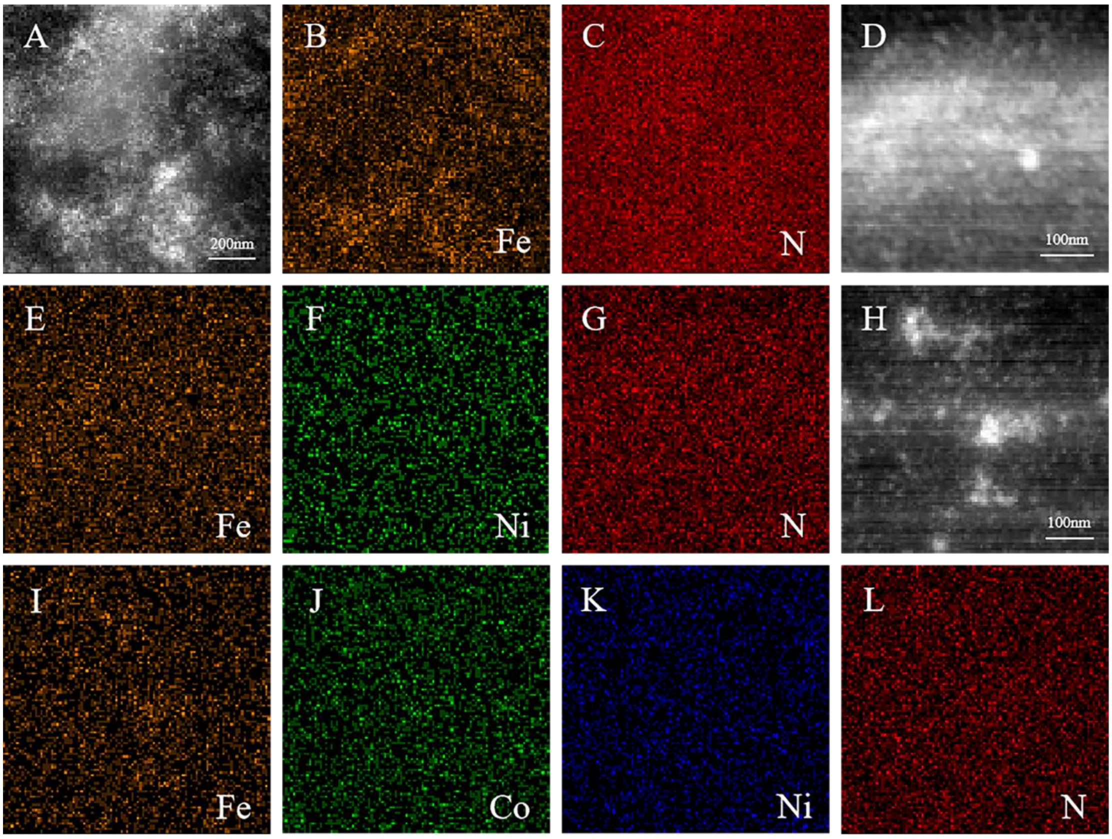

Figure S1. EDS mapping of (A–C) PB, (D–G) Ni-PB and (H–L) Co-Ni-PB.

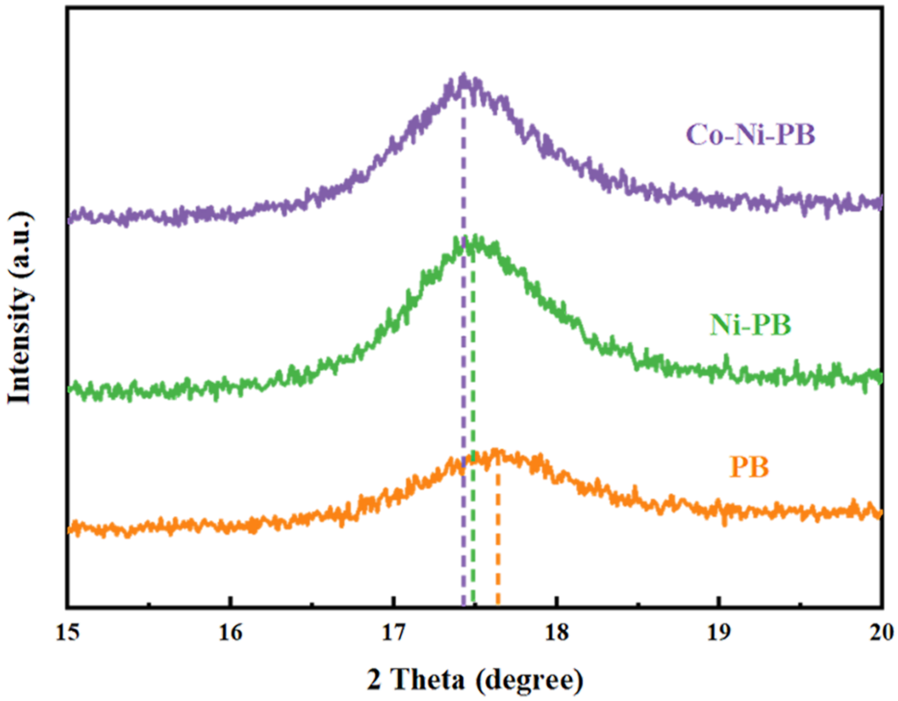

Figure S2. The enlarged view of XRD pattern.

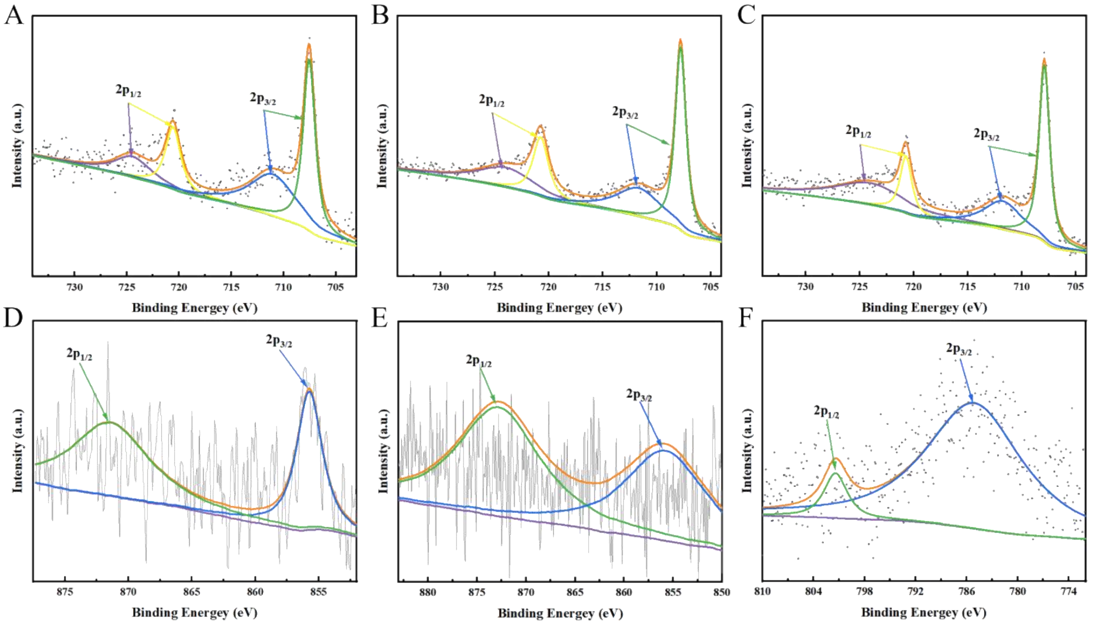

Figure S3. Fe 2p XPS spectra of (A) PB, (B) Ni-PB and (C) Co-Ni-PB. Ni 2p XPS spectra of (D) Ni-PB and (E) Co-Ni-PB. Co 2p XPS spectra of (F) Co-Ni-PB.

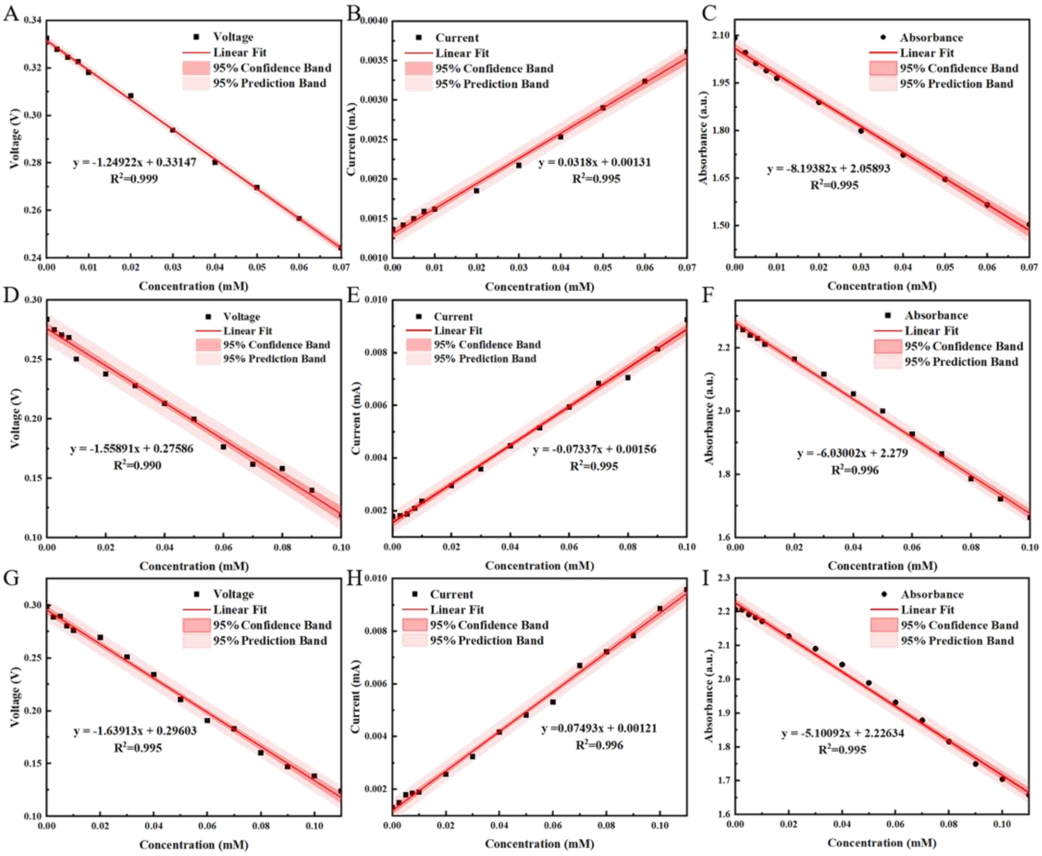

Figure S4. Calibration curves of OCV, CA and UV absorption of (A–C) PB, (D–F) Ni-PB and (G–I) Co-Ni-PB.

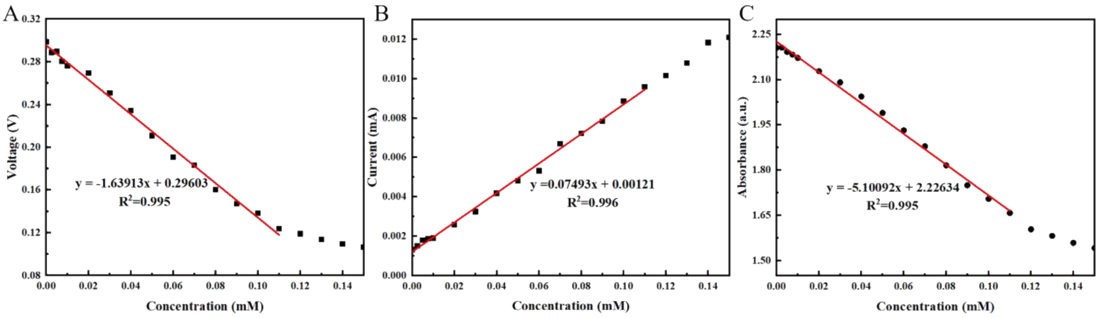

Figure S5. OCV, CA and UV absorption of Co-Ni-PB under the 0~0.15mM concentration of AA.

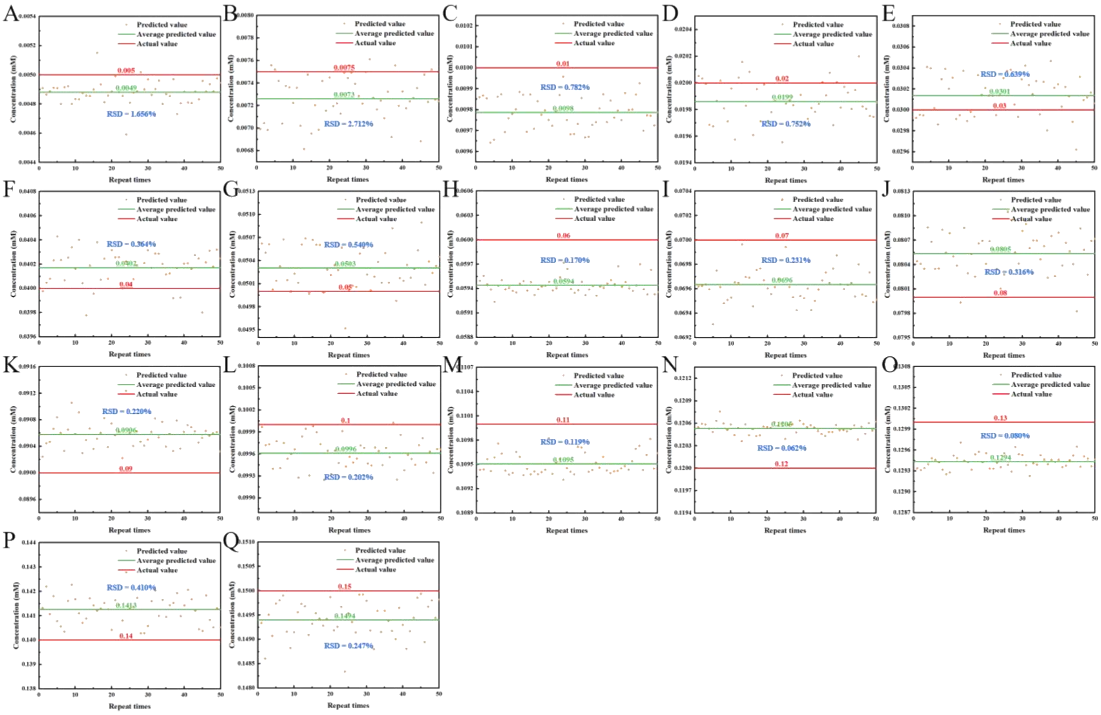

Figure S6. 50 prediction results of BP ANN at different test concentrations.
